# Editorial: Molecular diagnosis and epidemiology of human pathogens

**DOI:** 10.3389/fmicb.2023.1329115

**Published:** 2023-11-20

**Authors:** Wafa Achour, Ons Bouchami, Arabella Touati

**Affiliations:** ^1^Laboratory Department, Centre National de Greffe de Moelle Osseuse, Tunis, Tunisia; ^2^Research Laboratory, Microbiology of Children and Immunocompromised, (LR18ES39), Faculty of Medicine of Tunis, University Tunis El Manar, Tunis, Tunisia; ^3^Laboratory of Human Microbiota – Xenobiotics Interactions, Instituto de Tecnologia Química e Biológica António Xavier, Universidade Nova de Lisboa (ITQB-NOVA), Oeiras, Portugal; ^4^CHU de Bordeaux, Bacteriology Department, National Reference Center for Bacterial Sexually Transmitted Infections, F-33000, Bordeaux, France

**Keywords:** clinical medicine, infectious diseases, molecular diagnostics, molecular epidemiology, microbiology

In the past two decades, the expansion of Medical Microbiology is essentially due to the great advances in related fields such as immunology, genetics, bioinformatics, science, technology, engineering, and mathematics. This Research Topic provides an updated core of basic knowledge critical to clinical practice in medicine. Each article deals with an important human pathogen. It provides an update of the current state-of-the-art scientific knowledge applied to molecular diagnosis and epidemiology of human pathogens.

Integrative review, which highlights advances in molecular diagnosis of human pathogens, helps researchers, and practitioners to access and scrutinize the fast expanding knowledge. Cao et al. exposed microbiological and molecular biological diagnostics for tuberculous meningitis. Xiang et al., demonstrated, in a meta-analysis, that metagenomic next-generation sequencing had good specificity but moderate sensitivity for the early diagnosis of tuberculous meningitis. These articles deal with the difficult microbiological diagnosis of tuberculous meningitis, the most severe form of extra-pulmonary tuberculosis.

New scientific knowledge and understanding make new applications possible. Zheng et al. confirmed that hepatocyte growth factor plus adenosine deaminase is an excellent biomarker in tuberculous pleural effusion patients. In the field of tuberculosis, the World Health Organization has proposed the development of a diagnostic biomarker or triage biomarker among the high-priority target product. However, the development of a universal biomarker that diagnoses tuberculosis disease in both adults and children, pulmonary and extrapulmonary tuberculosis, and in varying stages of immunosuppression is challenging. Zhao et al. proposed a sensitive and specific digital droplet Polymerase Chain Reaction (PCR) assay for the detection of *Mycoplasma pneumoniae*. Digital Droplet PCR, known as the third generation of quantitative PCR, enables the exact quantification of nucleic acid targets within a sample. In contrast to real-time PCR, it relies on analysis of the endpoint of the PCR. Its capability to accurately detect and quantify low abundant targets has led to its fast-growing applications in detection of different pathogens. Chen et al. reported a novel molecular diagnostic assay named reverse transcription loop-mediated isothermal amplification combined with a visual gold nanoparticle-based lateral flow assay that can be eventually used as a point-of-care diagnostic tool for HIV-1 detection in clinical settings. Pollak et al. developed three rapid Nipah virus molecular diagnostic tests based on reverse transcription recombinase-based isothermal amplification coupled with lateral flow detection. In the last decade, many methods for the sequence-specific detection of Loop-mediated isothermal amplification have emerged as an important diagnostic tool. Indeed, rapid, and inexpensive diagnostic tests are necessary to control spread of disease in endemic settings where sophisticated laboratories may not be available.

Next-generation sequencing technologies are increasingly available in clinical microbiology laboratories. Their main applications are: whole genome sequencing, targeted metagenomics sequencing, and shotgun metagenomics sequencing. These applications are used for microbial identification, antimicrobial resistance genes detection and epidemiologic tracking of organisms. Yao et al. found that blood nanopore targeted sequencing can detect infection in deceased donors earlier and more accurately than bloodculture, which could raise the donation conversion rate. Sun et al. suggested that 16S *rRNA* gene next-generation sequencing is more suitable than aerobic culture for identification of polymicrobial pancreatic infections in severe and critical acute pancreatitis patients. Huang et al. concluded that PCR-based targeted next-generation sequencing can effectively identify periprosthetic joint infection pathogens and may provide information on drug resistance, while it is superior to metagenomic next-generation sequencing in terms of cost and turnaround time. Jaworska et al. found that urobiome composition in patients undergoing dialysis and in kidney transplanted patients is better characterized by amplicon sequencing than classical microbiology methods. Currently, the application of next-generation sequencing is mainly limited to academic or reference laboratories. Major decision-making regarding technologies, operational models, infrastructure, human resources, and professional expertise is needed before the widespread implementation of next-generation sequencing in clinical laboratories.

Nucleic acid amplification and next generation sequencing techniques are the most widely used methods in pathogen detection. These methods have become the gold standard as they detect even a few nucleic acid copies. However, they have numerous limitations such as complex thermal cycle process, complex primer design of PCR, unstable single base resolution of loop-mediated amplification and deep knowledge of biology to analyze data generated by next-generation sequencing. CRISPR-Cas-based method, a new generation of gene editing technology, may be an interesting approach. Ren et al. developed a sensitive and specific PCR-CRISPR/Cas13a method for *Mycobacterium tuberculosis* detection in sputum, bronchoalveolar lavage fluid and pus samples. Wang et al. developed a Cas12b-based one-pot platform by integrating isothermal amplification and CRISPR detection into one step, named CDetection.v2, enabljng the detection of SARS-CoV-2 in 30 min. Zhu et al. described latest advances in nucleic acid detection methods for SARS-CoV-2, in particular, biosensors and clustered regularly interspaced short palindromic repeats (CRISPRs)-based diagnostic systems. Emerging CRISPR technologies have been widely applied in combination with isothermal amplifications for SARS-CoV-2 detection. Their applications are still limited due to many reasons: immaturity of the CRISPR technology, incomplete auxiliary instruments and reagents, unconfirmed advantages over PCR (such as cost, stability, and convenience).

The pathogenesis and the epidemiology of microorganisms that colonize or infect humans had practical applications in treatment and prevention of diseases (infectious diseases, infection-related cancer, dysbiosis related diseases, etc.). Regarding infection-related cancer, Burassakarn et al. reported that the overall prevalence of HPV DNA was statistically associated with an increased risk of esophageal cancers. Seyoum et al., found that the presence of high-risk HPV, irrespective of genotypes, is highly correlated with cervical cell abnormalities. The distribution of HPV genotypes varies across continents, countries, and even within a single country. Morover, the current vaccines confer a limited cross-protection. Therefore, it is essential to generate scientific data concerning HPV prevalence, genotype distribution, cytological profile and associated factors in different populations in order to predict the efficacy of current vaccines and to develop a new vaccine strategy. Concerning dysbiosis related diseases, Li et al., based on 16S rRNA gene sequencing, had preliminary confirmed that dysbiosis occurs in patients susceptible to pathological scars. Growing evidence supports that gut microbial dysbiosis can promote the development and progression of different diseases via the interaction between gut microbiota and host. Deciphering the functional relationships in this symbiotic ecosystem, beyond the microbial DNA contents, permits a more comprehensive analysis.

Typing of causative pathogens is important to trace pathogens and to study microbial population dynamics which are necessary in infection prevention and control. Miellet et al. concluded that molecular testing of culture-enriched saliva samples improves the sensitivity of overall surveillance of pneumococcal carriage in children and adults. Jacqueline et al. recommended the analysis of the non-coding region MF-NCR to be added to N450 sequencing for measles molecular surveillance during post-elimination phase. Shin et al. provided a genotype fingerprinting method for recurrence tracing of heterogeneous *Mycobacterium intracellulare*. Although sequence-based typing methods offer new prospects for improving the resolution and comparability of typing systems for public health applications, the typing process in diagnostic laboratories remains laborious and time-consuming.

Genomic microbial identification is used in the diagnosis and monitoring of infectious diseases within the One Health concept. Ergunay et al. found that, in field-collected ticks, metagenomic nanopore sequencing is better than broad-range and nested amplification in virus detection and diversity investigation. Indeed, metagenome sequencing can be employed for bio- or xeno-surveillance, where blood-sucking arthropods are used as sentinels to screen pathogens, that may threaten the health of wildlife, livestock, and humans. Nemati et al. described rapid, selective, and easy-to-use biosensor and nanobiosensor technology, developed for early detection of common waterborne protozoa. Smart biosensing platforms play an extremely significant role. Electrochemical biosensors and electroanalytical techniques are adapted to point-of-care testing. Therefore, they are very useful in diagnosing, predicting and controlling infectious diseases epidemics or pandemic.

The pace and sophistication of advances in all scientific disciplines applied to medical microbiology become a challenge due to lack of expertise to analyze fast-growing data and to translate them in clinical practice and medical education. They call for a comprehensive and integrative understanding of the overall knowledge development in the different related fields.

In summary, this Research Topic deals with epidemiology, pathophysiology and diagnosis of important human pathogens. These pathogens have been investigated essentially in humans but also in vectors and in water. Adopting the One Health offers a powerful approach for improving human, animal, and environmental health and developing multi-level resilience across countries. Moreover, this topic presented affordable techniques for the diagnosis of epidemic pathogens (SARS-CoV-2, Nipah virus, *Mycobacterium tuberculosis*). Sustainable global and regional research and development networks are essential for pandemic preparedness efforts including strategies for sharing technology, data and knowledge to facilitate better access to affordable diagnostic, therapeutics and vaccines worldwide, particularly in developing countries for future pandemics.

## Author contributions

WA: Writing—original draft, Writing—review & editing. OB: Writing—review & editing. AT: Writing—review & editing.

